# Barriers to early diagnosis and treatment of oral cancer in countries in Africa: a scoping review

**DOI:** 10.1186/s12903-025-07072-1

**Published:** 2025-11-04

**Authors:** Mennatollah Nagy Sharkawy, Mohammed Sherif Amin, Moréniké Oluwátóyìn Foláyan

**Affiliations:** 1 Department of Pediatric Dentistry and Dental Public Health, School of Dentistry, Newgiza University, Giza, Egypt; 2 The Africa Oral Health Network (AFRONE), Alexandria University, Alexandria, Egypt; 3 Department of Clinical Infection, Microbiology and Immunology, University of Liverpool, Liverpool, UK; 4 Department of Paediatric Dentistry and Dental Public Health, Faculty of Dentistry, Ain Shams University, Ain Shams, Egypt; 5 Department of Child Dental Health, Obafemi Awolowo University, Ile Ife, Nigeria

**Keywords:** Cancer, Africa, Health system strengthening, Oral health promotion, Socio-ecological model

## Abstract

**Background:**

In Africa, the high mortality associated with oral cancer is driven by multiple factors. This ScR aims to map the existing evidence on the barriers to early diagnosis and treatment of oral cancer in Africa.

**Methods:**

A search was run between October and November 2024 in four electronic databases: Web of Science, PubMed, Scopus, and Cochrane Central Register of Controlled Trials (CENTRAL). A combination of the terms of the context was employed, which included the 54 African countries, and the concept, including a range of terms for oral cancer. Qualitative and quantitative primary studies were included, with eligibility determined by title and abstract screening followed by full-text screening. The data extracted were analysed using a thematic synthesis approach, and the themes were derived from the Socio-Ecological Model.

**Results:**

Eight studies met the eligibility criteria. The studies were hospital-based, descriptive cross-sectional studies conducted in six countries in East, West, and Southern Africa between 1999 and 2024. Common barriers to both early diagnosis and treatment of oral cancer include reliance on traditional medicine, lack of awareness about oral cancer symptoms, financial constraints, and the inability to forego daily income. At the organizational level, shared barriers include inefficient referral systems, long waiting times, and inadequate healthcare infrastructure. Unique factors for barriers to early diagnosis include rural residence, self-medication behaviours, low education levels, lack of public awareness, inadequate dentist training, frequent misdiagnoses, and poverty. In contrast, unique barriers to treatment involve negligence, fear of treatment, challenges in obtaining work leave, delays in clinical action, geographic challenges, long waits for histopathology results, and shortages of medications, beds, and functional equipment. Regional variations in barriers to early oral cancer diagnosis and treatment in Africa were observed, including gender related issues in West Africa.

**Conclusions:**

There are multiple-level barriers to the early diagnosis and treatment of oral cancer in countries in Africa. Addressing these barriers requires the combined efforts of multiple stakeholders and interventions tailored to regional needs to improve oral cancer outcomes and foster equitable healthcare access. Gender sensitive approaches to address caregiving burdens in Senegal are vital. Crucially, evidence is limited, so more studies must be conducted, especially in Northern and Central Africa, to address these barriers. Longitudinal and mixed-methods studies are also recommended to better understand how those barriers evolve.

## Introduction

 The global burden of cancers is rising, reaching 20 million incident cases in 2022 [1]. Oral cancers, including those affecting the lips and the tongue, are among the most common cancers globally [2]. An estimated 389,485 new cases of oral cancers and 188,230 deaths occurred in 2022 [2]. Although the burden of oral cancer in Africa is notably lower than in other regions, such as Asia [3], under-detection is likely masking the actual burden [4].

Multiple barriers hinder the timely diagnosis and treatment of oral cancers. These barriers include intrapersonal factors such as poor oral health knowledge; interpersonal factors such as cultural barriers, organizational factors related to the healthcare systems, and policy-level factors such as poverty [5]. In Africa, the rise in oral cancer burden is driven by multiple factors like limited access to healthcare, fragile healthcare infrastructure, limited financial resources [6], and poor public awareness of the early symptoms and risk factors for oral cancers [7]. As a result, oral cancers in Africa are often detected in advanced stages, with between 81% and 83% of the cases present with stage IV of the disease [8, 9]. Late diagnosis is associated with poorer prognosis, high treatment costs, and increased mortality [10]. Thus, early detection and treatment are crucial in improving outcomes [10].

Across Africa, the health care systems face multiple challenges, including insufficient human resources, inadequate healthcare financing, poor infrastructure, and ineffective leadership and management [11]. Furthermore, these systems do not prioritize patient education, and healthcare services are centralized, leading to underserved rural areas [12]. Corruption is also a significant challenge in the African health care systems at the governmental, hospital, and health care provider levels, which negatively impacts cancer care [13].This is reflected in the insufficient number of cancer treatment facilities, which fail to meet the needs of the African population, and the low per capita healthcare expenditure [14].

Previous reviews exploring the barriers to early detection and treatment of oral cancer had predominantly focused on Southeast Asian countries [6, 7], where oral cancer incidence is driven by region-specific factors such as high rates of tobacco and betel quid consumption [15]. The epidemiological and socio-cultural landscape of oral cancer in Africa differs significantly from that of Southeast Asia. Infectious agents (e.g., Human Papillomavirus and HIV), dietary habits, and traditional practices contribute to the aetiology of oral cancer in Africa [16], giving the region a distinct epidemiological profile from Southeast Asia. The barriers to early detection and treatment of oral cancer may also be unique to countries in Africa. Therefore, there is a critical need to address this gap in the literature by specifically exploring the barriers to early detection and treatment of oral cancer in the African context. The objective of this ScR is to map the existing evidence on the barriers to early diagnosis and treatment of oral cancer in Africa.

## Methods

Our ScR was conducted in adherence to the Joanna Briggs Institute (JBI) guidelines [17] for conducting scoping reviews, and reported in accordance with the Preferred Reporting Items for Systematic reviews and Meta-Analyses extension for Scoping Reviews statement (PRISMA ScR) [18]. The PCC (population, concept, and context) mnemonic was employed to guide the review. The population referred to individuals in Africa at risk of or diagnosed with oral cancers. The concept was the barriers to the early diagnosis and treatment of oral cancers. The context was limited to studies conducted within the geographical and healthcare settings of 54 African countries recognized by the United Nations [19].

### Information sources and search strategy

Two independent reviewers (M.S. and M.A.) ran the search in four electronic databases: Web of Science, PubMed, Scopus, and Cochrane Central Register of Controlled Trials (CENTRAL). The searches were first conducted on 6 October 2024 and were repeated on 4 November 2024 to identify any newly published studies.

A combination of the terms of the elements, context, and concept was employed. The context included the 54 African countries, while the concept included a range of terms for oral cancer, including “oral cancer,” “mouth cancer,” “mouth neoplasm,” “mouth tumor,” “oral neoplasm,” “oral tumor,” “oropharyngeal cancer,” “oropharyngeal neoplasm,” “tongue cancer,” “palatal cancer,” “cheek cancer,” “buccal cancer,” “floor of mouth cancer,” “squamous cell carcinoma,” and related variations such as “malignanc*.“These terms were identified based on the keywords and controlled vocabulary (e.g., MeSH or emtree) identified during initial literature scoping, along with expert consultation. Boolean operations, truncation, and wildcards were used, and the final search builder of each database can be found in Appendix 1. In addition to the electronic search, a manual citation search was performed across the retrieved articles. The searching process was presented in the PRISMA flow diagram [20].

### Eligibility criteria

Studies were included in the scoping review if they explicitly investigated barriers to the early diagnosis and treatment of oral cancer within any of the 54 African countries recognized by the United Nations. Studies that examined multiple cancer types were eligible only if they explicitly reported barriers related to oral cancers. Oral cancers were defined by the ICD-10 codes C00-C14.

Qualitative and quantitative primary studies were considered eligible for inclusion in the study. No restrictions were placed on the publication date to ensure the inclusion of all relevant evidence.

To ensure the relevance of the evidence base, reviews of any type, including systematic, scoping, and rapid reviews, as well as editorials, books, and opinion pieces, were excluded. Publications written in languages other than English were also excluded, given resource constraints for translation and analysis. Furthermore, studies that addressed barriers to early diagnosis and treatment of oral cancer as a secondary or peripheral conclusion to other primary research objectives were not considered eligible for inclusion. Articles for which the full text was inaccessible, despite reasonable efforts to obtain them, were also excluded.

### Data screening

All articles retrieved from the initial database search were imported into EndNote reference management software (EndNote 20), where duplicates were systematically identified and removed. Following this, a two-stage screening process was conducted in accordance with the predetermined inclusion and exclusion criteria. The first stage involved title and abstract screening, which was performed independently by two reviewers (M.S. and M.A.) to minimize bias and enhance reliability. Any discrepancies or conflicts arising during this stage were resolved through consensus. When consensus was not reached, a third reviewer (M.O.F) made the determination.

In the second stage, full-text screening was conducted to ensure the eligibility of articles for final inclusion. Each of the two reviewers (M.S. and M.A.) initially screened half of the articles independently, followed by an alternating review process to cross-check the decisions. This alternating approach ensured that all articles were reviewed by both reviewers. Articles for which eligibility remained uncertain after this step were revisited collectively and assessed collaboratively to reach a final decision. This process was followed by a manual citation search of the reference lists of all included articles to identify any potentially relevant studies that might not have been captured during the initial database search.

### Data charting

The charting process began with documenting the title of each article to provide a clear reference point for all included studies. The publication date was extracted to contextualize the findings within the timeline of research development in this field. The geographical setting of each study, specifically the country in which the study was conducted, was documented to capture the regional representation and understand the diversity of contexts within Africa. This information was crucial for identifying location-specific barriers to the early diagnosis and treatment of oral cancer.

In addition, the study population was described in detail, noting characteristics such as demographic and clinical factors that could influence the barriers investigated. The data collection methods employed were recorded. This included qualitative, quantitative, or mixed methods approaches, as well as specific tools and techniques used to gather information. Furthermore, the information on the sample size of each study was extracted to reflect the scale of the research and its potential impact on the generalizability of the findings. Where available, the age of the study population was documented to explore age-related disparities in barriers to early diagnosis and treatment. This demographic detail provided an additional layer of analysis, particularly in understanding how different age groups might experience challenges differently.

The primary focus of the charting process was on identifying and categorizing barriers to the early diagnosis and treatment of oral cancer. These were reported separately to maintain clarity and specificity in the analysis. In cases where studies did not report on either early diagnosis or treatment barriers, the respective fields were left blank in the characteristics table. The data charting process was conducted independently by two reviewers to minimize bias and enhance reliability. Discrepancies in data extraction were resolved through discussions and consensus.

#### Data analysis

To summarize the characteristics of the included studies, the analysis documented key details such as sample size, geographical distribution, study designs, and publication timelines. Where applicable, data were stratified by geographical region (Western, Eastern, Northern, Southern, and Central Africa) to explore contextual differences. The data extracted were analysed using a thematic synthesis approach, which is well-suited for identifying patterns and summarizing qualitative and mixed-methods data across diverse contexts. The themes were derived from the Socio-Ecological Model [21, 22]**.** The SEM considers multiple levels of influence on health outcomes, ranging from individual to systemic levels. Patient-related barriers (financial constraints, rural settlement, low education) and healthcare-related barriers (misdiagnosis, long waiting times, inadequate training) extracted from the literature were re-classified as individual, interpersonal, organizational, and policy levels. Thus, the SEM is both comprehensive and appropriate for capturing the complex nature of barriers that hinder the timely diagnosis and treatment of oral cancer [5]. In the African context specifically, these challenges are multifaceted, ranging from individual-level to systemic barriers [11]. Therefore, applying the SEM provides a structured presentation of these barriers, making it easier to communicate findings with the multiple stakeholders, including policy makers, healthcare providers, and public health practitioners.

## Results

**Fig. 1:**
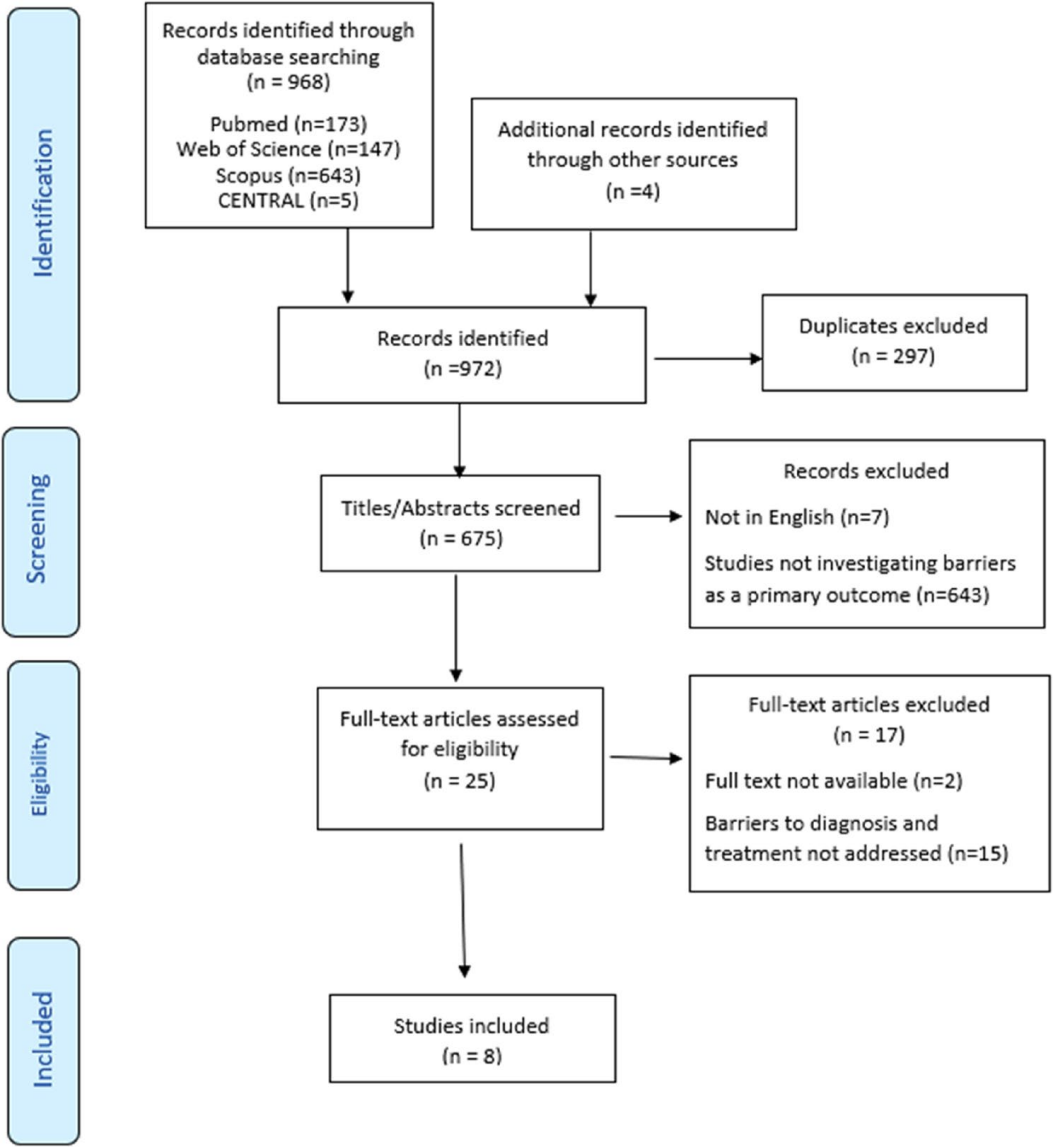
Preferred Reporting Items for Systematic Reviews and Meta-Analyses (PRISMA) flow diagram for the scoping review process

As presented in the flow chart (Fig. 1). The database and hand searches retrieved 972 records. After duplicate removal, 675 abstracts were reviewed, and only 25 records were eligible for full-text screening. Following the full text screening, 15 articles did not address the barriers clearly, and two articles were not present in full text. Finally, eight articles were included in our review.

### Characteristics of the studies

Table 1 shows the characteristics of the eight studies included in the scoping review. The studies were conducted between 1999 and 2024; six of the eight studies were conducted after 2015. Five studies were conducted in East Africa: in Tanzania [23, 24] and Kenya, [25, 26] respectively, and one in Sudan [27]. Two studies were conducted in West Africa: one in Senegal [28] and one in Nigeria [29], respectively. One study was conducted in Botswana, Southern Africa [30]. No studies from Northern and Central Africa were identified. In addition, the sample sizes are generally small, and all the studies are hospital-based.

**Table 1:** Characteristics of the included studies

Study	Year	Country (Region)	Aim of the study	Study Design	Study Population	Data collection method	Sample Size
Kimario et al. [23]	2024	Tanzania (East Africa)	To determine the stage of head and neck malignancies at presentation at Bugando Medical Centre and the factors that contributed to the patients’ presentation at that stage.	Cross-sectional	Hospital-based study 35 males, 25 females 12–89 years old	Questionnaire	60 patients
Msolla et al. [24]	2019	Tanzania (East Africa)	To determine the reasons for late reporting among patients with oral and maxillofacial tumours and tumour-like lesions at the Muhimbili National Hospital.	Cross-sectional	Hospital-based study 62 males, 82 females 1–90 years old	Questionnaire	144 patients
Augustine [25]	2021	Kenya (East Africa)	To determine the factors responsible for the delay in diagnosis and treatment of patients with oral cancer.	Cross-sectional	Hospital-based study 43 males, 28 females 14–80 years old	Questionnaire	71 patients
Onyango and Macharia [26]	2006	Kenya (East Africa)	To determine the causes of late presentation of head and neck cancer at Kenyatta National Hospital.	Cross-sectional	Hospital-based study 34 males,10 females 20–89 years old	Patient interviews, review of health records	44 patients
Ahmed &Naidoo [27]	2017	Sudan (East Africa)	To determine dentists’ knowledge, attitudes, and practices in the prevention and early detection of oral cancers, in Khartoum State. To evaluate continuing education needs of oral cancer risk factors and clinical diagnostic skills.	Cross-sectional	Hospital-based study 36 males, 77 females	Questionnaire	113 dentists
Beaudoin et al. [28]	2022	Senegal (West Africa)	To identify the barriers to care for patients with head & neck cancer	Mixed methods	Hospital based study 24 males, 9 females 18–84 years old	Questionnaire and semi-structured interview	33 patients
Oji [29]	1999	Nigeria (West Africa)	To highlight the factors contributing to the delays in presentation and treatment of orofacial tumours in Nigeria	Case series	Hospital-based study 80 males, 48 females	Review of case notes	128 patients
Precious et al. [30]	2022	Botswana (South Africa)	To identify risk factors for presenting with advanced-stage oral cancer	Retrospective cohort analysis	Hospital-based study 172 males, 46 females 47–63 years old	Data extraction from hospital records and databases	218 patients

The number of study participants was 811, with the number of study participants in each study ranging from 33 patients [28] to 218 patients [30]. The studies included 325 females and 486 males. Five of the studies were cross-sectional in design [23–27]. The others were a cohort study [30], case series [29], and a mixed-methods study [28]. The studies were descriptive [23–30] and generated data using questionnaires [23–25, 27, 28] in combination with extracting data from hospital records [26, 29, 30] or semi-structured interviews [28]. All studies were hospital-based.

One study investigated the stage of head and neck cancer (HNC) at the time of clinical presentation [23]. Another explored the barriers to HNC care [28], while other studies focused on the risk factors associated with late presentation and treatment of HNC [24–26, 29, 30]. One study in Sudan explored the dentists’ knowledge, attitudes, and practices regarding the early detection and prevention of oral cancer from the dentists’ perspectives [27].

### Barriers to early diagnosis

Table 2 shows that the individual-level barriers to early diagnosis of oral cancer were rural settlement [23], use of traditional medicine [23, 28, 29], and self-medication behaviours [25]. Additionally, lower levels of education [23, 29] and a lack of proper patients’ knowledge and awareness regarding oral cancer symptoms acted as barriers to early diagnosis [25, 28, 30]. Furthermore, financial constraints due to costly clinical consultations and hospital treatments prevented timely diagnosis [25, 26, 28, 29]. Also, the fear of losing a job or a daily income presented individual-level barriers to early diagnosis [28].

**Table 2:** Barriers to early diagnosis and treatment of oral cancer

Study	Barriers to early diagnosis	Barriers to treatment
Kimario et al. [23]	**Individual-level factors** • Rural settlement • Use of traditional medicine • Lower levels of education **Interpersonal-level factors** • Influence of family or community norms supporting traditional medicine **Organizational-level factors** • Attending the dispensary for the first visit rather than higher levels of healthcare	
Msolla et al. [24]	**Organizational-level factors** • Remote healthcare facility	**Individual-level factors** • Cost • Use of traditional medicine • Fear of treatment • Negligence **Interpersonal-level factors** • Difficulty in obtaining permission from work
Augustine [25]	**Individual-level factors** • Lack of patient awareness regarding cancer symptoms. • Self-medication behaviors. • Financial barriers. **Organizational-level factors** • Delayed clinical diagnosis • Prolonged referral times. • High treatment cost	**Individual-level factors** • High costs of treatment **Interpersonal-level factors** • Lack of prompt clinical action **Organizational-level factors** • Inefficiencies in the referral system • Treatment delays at referral centers •Long wait times for histopathology results
Onyango and Macharia [26]	**Individual-level factors** • Financial constraints impacting access to timely care **Organizational-level factors** • Lack of public awareness regarding cancer symptoms • Multiple referrals before reaching a tertiary hospital	**Individual-level factors** • Low cancer awareness **Organizational-level factors** • Delay in diagnosis at primary healthcare levels • Lack of direct referral systems, leading to extended pathways to care • Inadequate facilities
Ahmed &Naidoo [27]	**Organizational-level factors** • Inadequate training of dentists to detect oral cancer lesions	
Beaudoin et al. [28]	**Individual-level factors** • Expensive clinical consultation • Preference for traditional medicine • Misunderstanding of signs and symptoms of head and neck cancer • Fear of losing a job • Inability to afford to lose a day’s income **Interpersonal-level factors** • Family and children’s duties (as caregiving responsibilities may limit access to healthcare) **Organizational-level factors** • Expensive travel costs to healthcare facilities • Remote healthcare facilities • Long waiting times for both presentation and referral • Lack of referral systems • Misdiagnosis at healthcare facilities • Unavailable means of transportation to healthcare facilities	**Individual-level factors** • Expensive clinical consultation • Preference for traditional medicine • Misunderstanding of signs and symptoms of head and neck cancer • Fear of losing a job • Inability to afford to lose a day’s income. **Organizational-level factors** • Institutional barriers within healthcare facilities, including defective radiotherapy equipment, shortages of medications, and lack of available beds.
Oji [29]	**Individual-level factors** • Preference for traditional medicine. • High costs of hospital treatments. • Lack of education **Organizational-level factors** • Limited access for rural patients • Long distances from healthcare facilities. **Policy-level factors** • Poverty	**Individual-level factors** • Preference for non-hospital treatments such as traditional medicine. • Financial constraints. **Organizational-level factors** • Geographic barriers.
Precious et al. [30]	**Individual-level factors** • Lack of awareness of oral cancer symptoms. • Difficulty accessing care for hidden tumors in less visible anatomical sites. **Interpersonal-level factors** • Socioeconomic challenges. **Organizational-level factors** • Limited access to healthcare in economically disadvantaged districts. .	

The interpersonal factors included socioeconomic challenges, family responsibilities [28, 30], and the influence of family or community norms supporting traditional medicine [23]. The organizational-level barriers were remote, inaccessible healthcare facilities [24, 28–30], lack of proper referral systems [23, 25, 26, 28], long waiting times [28], and expensive treatment costs [25, 28]. Also, a lack of public awareness [26], inadequate training of dentists and misdiagnosis contributed to the delayed diagnosis of oral cancer [27, 28]. The only policy-level factor identified in the publications was poverty [29].

### Barriers to early treatment

Table 2 shows that the individual-level barriers to early treatment of oral cancer were financial constraints, presented in the high costs of treatment and clinical consultation [24, 25, 28, 29]. In addition, the lack of patients’ awareness of oral cancer [26, 28], negligence [24], fear of treatment [24], and a preference for traditional medicine [24, 28, 29] were among the individual-level barriers. Also, the inability to lose one’s daily income was a barrier to early treatment [28].

The interpersonal-level factors were the inability to take an absence of permission from work [24] and a lack of prompt clinical action [25]. The organisational-level factors were delayed diagnosis [26], inefficient referral systems [25, 26], geographic barriers [29], and long wait times for histopathologic results [25]. In addition, inadequate health care facilities with shortages of medication, beds, and defective equipment presented organizational-level barriers to early treatment [26, 28]. No policy-level barriers were identified.

### Regional differences and similarities in barriers to early diagnosis and treatment of oral cancer

In East Africa, barriers to early diagnosis and treatment are shaped by challenges in navigating healthcare systems and societal norms. Tanzania and Kenya share an emphasis on inefficiencies within referral systems [25, 26], while Sudan uniquely highlights inadequate training of healthcare providers as a factor hindering early detection [27]. In addition, Tanzania identified interpersonal challenges, such as workplace dynamics, impeding timely treatment [24].

In West Africa, structural and organizational barriers are the major barriers. Senegal reports on severe healthcare infrastructure deficits, including defective equipment, medication shortages, and ineffective referral systems [28]. Furthermore, Senegal faces significant interpersonal challenges, with caregiving responsibilities often falling disproportionately on women, acting as a critical obstacle to seeking care [28]. Nigeria, on the other hand, underscores the pervasive impact of poverty and geographic inaccessibility, particularly for rural populations, as central barriers [29].

In Southern Africa, the study from Botswana highlights how economic inequality and geographic disparities shape barriers to care. Limited healthcare access in economically disadvantaged and rural areas creates significant obstacles to early diagnosis [30]. Unlike East and West Africa, where traditional medicine plays a prominent role [23–25, 29], the study from Southern Africa (Botswana) placed greater emphasis on financial barriers and equitable access to healthcare services [30].

## Discussion

The current scoping review of eight hospital-based, descriptive cross-sectional studies conducted in East, West, and Southern Africa examines barriers to early diagnosis and treatment of oral cancer. Common factors that serve as barriers to both early diagnosis and treatment of oral cancer include reliance on traditional medicine, lack of awareness about oral cancer symptoms, financial constraints, and the inability to forego daily income. At the organizational level, shared barriers include inefficient referral systems, long waiting times, and inadequate healthcare infrastructure. The review also identifies unique barriers to early diagnosis, such as rural residence and self-medication, and unique barriers to late treatment, including negligence and fear of treatment.

The study provides valuable insights into barriers to oral cancer care in Africa. It specifically addresses the African context, where oral cancer is under-researched, providing a regionally relevant evidence base. The review adhered to JBI and PRISMA-ScR guidelines, ensuring systematic and transparent processes for data collection, analysis, and reporting. In addition, the use of the SEM allows for a structured analysis of barriers across individual, interpersonal, organizational, and policy levels.

The study, however, had some limitations. The review includes studies from only three (East, West, and Southern Africa) of the five regions in Africa, limiting its generalizability across the continent. In addition, the studies collectively recruited few participants, which may not capture the full variability of barriers in different settings. All included studies were hospital-based, potentially excluding community-level insights and perspectives from patients who never reached healthcare facilities. Furthermore, the few studies span over two decades, during which healthcare systems and barriers may have evolved, making it challenging to generalize findings across time. In addition, as the review was limited to English-language publications, relevant literature in other languages (e.g., French in West Africa) may have been overlooked. Finally, there was a limited exploration of how policies impacted oral cancer diagnosis and treatment access. Despite these limitations, the study highlights several important findings. These findings provide an initial understanding of the multiple-level barriers affecting early treatment and diagnosis of oral cancer in countries in Africa, based on the currently limited evidence.

First, the findings on the barriers to early diagnosis and treatment of oral cancer in Africa feature systemic challenges that may hinder equitable access to healthcare. These barriers appear to be influenced by structural issues, limited funding, and inadequate infrastructure, often aggravated by the absence of enabling policies. A recurrent theme in this scoping review is that many underserved regions, especially rural and remote areas, experience significant healthcare gaps, primarily due to poor infrastructure. Structural barriers, such as a lack of healthcare facilities, limited diagnostic services, and difficulties in accessing care due to distance, seem to result from ineffective policy interventions [31].These factors could potentially contribute to healthcare inequities [32]. Addressing these challenges may require further investigation into how governments can better allocate resources to enhance healthcare infrastructure, especially in rural areas [33]. Moreover, the development of primary and specialized care facilities closer to underserved populations [34], as well as the use of mobile clinics and telemedicine [35] might offer opportunities to bridge gaps where physical infrastructure is insufficient.

Also, as identified by prior studies, healthcare costs, including consultation fees, diagnostic services, and treatment, remain prohibitive for many individuals in Africa [11]. These financial barriers are rooted in broader policy issues, such as insufficient public healthcare funding and the absence of subsidies or insurance mechanisms to mitigate costs [36]. Policies that fail to account for the economic vulnerabilities of rural and low-income populations exacerbate disparities in access to timely diagnosis and care [37]. Policy reforms should focus on introducing subsidies [37], expanding insurance coverage [38], and implementing universal health coverage (UHC) schemes [39] to alleviate financial burdens. UHC, however, needs to be complemented by concurrent investments in healthcare infrastructure and workforce development to improve healthcare access in rural areas [40].

Another systemic barrier identified is the issue of delayed and misdiagnosis resulting from inadequate investment in training and re-training of healthcare providers, particularly in continuing professional education. We identified that healthcare workers, including dentists, often lack the knowledge or skills required for early detection and management of oral cancer, resulting in delayed diagnosis [41]. This differs from the reports from countries in the global North, like Canada [42] and Brazil [43]. Patients welcome oral cancer screening and are willing to be informed about the signs of oral cancer [44]. Delayed diagnosis of oral cancer can be addressed by organising regular training programs, certifications, and workshops for healthcare providers, with an emphasis on early cancer detection and treatment. Partnerships with academic institutions and international organizations can enhance capacity-building initiatives.

Our study also identified that hospital inefficiencies, such as long waiting times and inadequate referral systems, further deter the timely diagnosis and treatment of oral cancer [23, 25, 26, 28]. These inefficiencies stem from weak institutional-level governance that fails to prioritize streamlined workflows and effective patient management systems. Healthcare institutions can improve their practices by implementing electronic referral systems, increasing staffing levels, and adopting lean management principles to optimize hospital operations. For Africa, where the number of oral health workers is a challenge, training mid-level healthcare workers and Community Health Workers to screen for oral cancer and refer for appropriate treatment may be a way to bridge workforce gaps, improve service delivery efficiency, and expand access to underserved populations.

Weak policies and practices on cancer prevention, including public education campaigns, are another major barrier. A lack of awareness about the symptoms of oral cancer and the importance of early diagnosis often leads to delayed care-seeking behaviour [45]. Public education on self-examination for oral cancer is feasible [46, 47]. A neglect of public health messaging and education as critical components of cancer prevention contributes to delayed diagnosis and treatment. Widespread public health campaigns that focus on raising awareness about oral cancer symptoms, prevention strategies, and the benefits of early diagnosis, by integrating these campaigns into existing health promotion initiatives, can amplify their reach and impact. In Africa, integrating oral cancer prevention and public education campaigns into the responsibilities of Community Health Workers presents an effective strategy for reducing delays in diagnosis and treatment. With proper training, Community Health Workers can conduct oral cancer screenings, raise awareness about early signs, and ensure timely referrals for appropriate treatment [48, 49].

The identified regional variations in barriers to early oral cancer diagnosis and treatment in Africa suggest the need for tailored strategies. East Africa faces challenges from healthcare system inefficiencies and societal norms where referral delays and reliance on traditional medicine hinder timely care. In West Africa, structural and socioeconomic obstacles dominate, with poverty, poor infrastructure, and caregiving responsibilities among women in Senegal, exacerbating disparities. Southern Africa is primarily affected by economic and geographic inequities, delaying access to care, with less emphasis on traditional medicine compared to other regions. Addressing these challenges requires localized interventions. Infrastructure upgrades and streamlined referral systems are vital in West and Southern Africa, while financial support programs are needed across all regions. Provider training, especially in Sudan, is essential to enhance early detection. Gender-sensitive approaches, such as those addressing caregiving burdens in Senegal, are also critical. These interventions, tailored to regional needs, can improve cancer outcomes and foster equitable healthcare access. Nevertheless, these regional variations should be interpreted with caution, given the small number of available studies. Further work is needed to explore whether such variations are consistent across other cancers and stigmatized health conditions in the African context.

This study revealed that the barriers to early diagnosis and treatment of oral cancer in Africa are complex and multifaceted, stemming from systemic deficiencies in infrastructure, funding, workforce training, public awareness, and individual-level challenges. The findings indicate that many individual-level issues are closely linked to broader systemic (macro) and organizational (meso) factors, which require targeted government investments in oral cancer-specific policies and programs. These policies and programs must adopt a cross-sectoral approach to address systemic challenges that impact both organizations and individuals, such as poverty, limited access to education, inadequate transportation systems, and restricted hospital access.

There are systemic issues, such as political instability in some regions, and economic policies that fail to prioritize healthcare funding, that create persistent barriers to implementing effective healthcare policies. Political commitment to health as a national priority is essential to be able to address the identified concerns about late diagnosis and treatment of oral cancers. When governments ensure stable healthcare financing and develop policies that protect healthcare budgets even in times of economic downturn or political instability, changes in the management of oral cancer may be observed.

## Conclusion

Oral cancer presents a high burden in African countries and requires special attention from multiple stakeholders. Barriers to the early diagnosis and treatment of oral cancer in African countries are multifactorial, and addressing those barriers can mitigate the burden of the disease and improve its prognosis. Community-based studies across diverse regions of Africa would help address the scarcity of research beyond hospital settings and capture broader patient perspectives. Larger samples could improve generalizability, while longitudinal approaches may clarify how barriers evolve. More recent evidence on current health care challenges, along with studies examining practical interventions, could further inform strategies to reduce these barriers in African contexts.

## Data Availability

No datasets were generated or analysed during the current study.

## Appendix

**Table:**

Database		Search builder	Hits	Date
PubMed	#1	“Oral cancer“[Title/Abstract] OR “Mouth cancer“[Title/Abstract] OR “Mouth neoplasm“[Title/Abstract] OR “Mouth tumor“[Title/Abstract] OR “Oral neoplasm“[Title/Abstract] OR “Oral tumor“[Title/Abstract] OR “Oropharyngeal cancer“[Title/Abstract] OR “Oropharyngeal neoplasm“[Title/Abstract] OR “Tongue cancer“[Title/Abstract] OR “Palatal cancer“[Title/Abstract] OR “Cheek cancer“[Title/Abstract] OR “Buccal cancer“[Title/Abstract] OR “Floor of mouth cancer“[Title/Abstract]		
	#2	“Africa“[Title/Abstract] OR “Algeria“[Title/Abstract] OR “Angola“[Title/Abstract] OR “Benin“[Title/Abstract] OR “Botswana“[Title/Abstract] OR “Burkina Faso“[Title/Abstract] OR “Burundi“[Title/Abstract] OR “Cabo Verde“[Title/Abstract] OR “Cameroon“[Title/Abstract] OR “Central African Republic“[Title/Abstract] OR “Chad“[Title/Abstract] OR “Comoros“[Title/Abstract] OR “Congo“[Title/Abstract] OR “Côte d’Ivoire“[Title/Abstract] OR “Democratic Republic of the Congo“[Title/Abstract] OR “Djibouti“[Title/Abstract] OR “Egypt“[Title/Abstract] OR “Equatorial Guinea“[Title/Abstract] OR “Eritrea“[Title/Abstract] OR “Eswatini“[Title/Abstract] OR “Ethiopia“[Title/Abstract] OR “Gabon“[Title/Abstract] OR “Gambia“[Title/Abstract] OR “Ghana“[Title/Abstract] OR “Guinea“[Title/Abstract] OR “Guinea-Bissau“[Title/Abstract] OR “Kenya“[Title/Abstract] OR “Lesotho“[Title/Abstract] OR “Liberia“[Title/Abstract] OR “Libya“[Title/Abstract] OR “Madagascar“[Title/Abstract] OR “Malawi“[Title/Abstract] OR “Mali“[Title/Abstract] OR “Mauritania“[Title/Abstract] OR “Mauritius“[Title/Abstract] OR “Morocco“[Title/Abstract] OR “Mozambique“[Title/Abstract] OR “Namibia“[Title/Abstract] OR “Niger“[Title/Abstract] OR “Nigeria“[Title/Abstract] OR “Rwanda“[Title/Abstract] OR “Sao Tome and Principe“[Title/Abstract] OR “Senegal“[Title/Abstract] OR “Seychelles“[Title/Abstract] OR “Sierra Leone“[Title/Abstract] OR “Somalia“[Title/Abstract] OR “South Africa“[Title/Abstract] OR “South Sudan“[Title/Abstract] OR “Sudan“[Title/Abstract] OR “Togo“[Title/Abstract] OR “Tunisia“[Title/Abstract] OR “Uganda“[Title/Abstract] OR “United Republic of Tanzania“[Title/Abstract] OR “Zambia“[Title/Abstract] OR “Zimbabwe“[Title/Abstract]	173	06/11/2024 07:24:31
	#3	#1 AND #2		
Web of Science	#1	(TI=((“Africa” OR “Algeria” OR “Angola” OR “Benin” OR “Botswana” OR “Burkina Faso” OR “Burundi” OR “Cabo Verde” OR “Cameroon” OR “Central African Republic” OR “Chad” OR “Comoros” OR “Congo” OR “Côte d’Ivoire” OR “Democratic Republic of the Congo” OR “Djibouti” OR “Egypt” OR “Equatorial Guinea” OR “Eritrea” OR “Eswatini” OR “Ethiopia” OR “Gabon” OR “Gambia” OR “Ghana” OR “Guinea” OR “Guinea-Bissau” OR “Kenya” OR “Lesotho” OR “Liberia” OR “Libya” OR “Madagascar” OR “Malawi” OR “Mali” OR “Mauritania” OR “Mauritius” OR “Morocco” OR “Mozambique” OR “Namibia” OR “Niger” OR “Nigeria” OR “Rwanda” OR “Sao Tome and Principe” OR “Senegal” OR “Seychelles” OR “Sierra Leone” OR “Somalia” OR “South Africa” OR “South Sudan” OR “Sudan” OR “Togo” OR “Tunisia” OR “Uganda” OR “United Republic of Tanzania” OR “Zambia” OR “Zimbabwe”))) OR AB=((“Africa” OR “Algeria” OR “Angola” OR “Benin” OR “Botswana” OR “Burkina Faso” OR “Burundi” OR “Cabo Verde” OR “Cameroon” OR “Central African Republic” OR “Chad” OR “Comoros” OR “Congo” OR “Côte d’Ivoire” OR “Democratic Republic of the Congo” OR “Djibouti” OR “Egypt” OR “Equatorial Guinea” OR “Eritrea” OR “Eswatini” OR “Ethiopia” OR “Gabon” OR “Gambia” OR “Ghana” OR “Guinea” OR “Guinea-Bissau” OR “Kenya” OR “Lesotho” OR “Liberia” OR “Libya” OR “Madagascar” OR “Malawi” OR “Mali” OR “Mauritania” OR “Mauritius” OR “Morocco” OR “Mozambique” OR “Namibia” OR “Niger” OR “Nigeria” OR “Rwanda” OR “Sao Tome and Principe” OR “Senegal” OR “Seychelles” OR “Sierra Leone” OR “Somalia” OR “South Africa” OR “South Sudan” OR “Sudan” OR “Togo” OR “Tunisia” OR “Uganda” OR “United Republic of Tanzania” OR “Zambia” OR “Zimbabwe”))	147	06/11/2024 11:43:00
	#2	(TI=((“Oral cancer” OR “Mouth cancer” OR “Mouth neoplasm” OR “Mouth tumor” OR “Oral neoplasm” OR “Oral tumor” OR “Oropharyngeal cancer” OR “Oropharyngeal neoplasm” OR “Tongue cancer” OR “Palatal cancer” OR “Cheek cancer” OR “Buccal cancer” OR “Floor of mouth cancer”))) OR AB=((“Oral cancer” OR “Mouth cancer” OR “Mouth neoplasm” OR “Mouth tumor” OR “Oral neoplasm” OR “Oral tumor” OR “Oropharyngeal cancer” OR “Oropharyngeal neoplasm” OR “Tongue cancer” OR “Palatal cancer” OR “Cheek cancer” OR “Buccal cancer” OR “Floor of mouth cancer”))		
	#3	#1 AND #2		
Scopus	(TITLE-ABS-KEY (“Oral cancer” OR “Mouth cancer” OR “Mouth neoplasm” OR “Mouth tumor” OR “Oral neoplasm” OR “Oral tumor” OR “Oropharyngeal cancer” OR “Oropharyngeal neoplasm” OR “Tongue cancer” OR “Palatal cancer” OR “Cheek cancer” OR “Buccal cancer” OR “Floor of mouth cancer”) AND TITLE-ABS-KEY (“Africa” OR “Algeria” OR “Angola” OR “Benin” OR “Botswana” OR “Burkina Faso” OR “Burundi” OR “Cabo Verde” OR “Cameroon” OR “Central African Republic” OR “Chad” OR “Comoros” OR “Congo” OR “Côte d’Ivoire” OR “Democratic Republic of the Congo” OR “Djibouti” OR “Egypt” OR “Equatorial Guinea” OR “Eritrea” OR “Eswatini” OR “Ethiopia” OR “Gabon” OR “Gambia” OR “Ghana” OR “Guinea” OR “Guinea-Bissau” OR “Kenya” OR “Lesotho” OR “Liberia” OR “Libya” OR “Madagascar” OR “Malawi” OR “Mali” OR “Mauritania” OR “Mauritius” OR “Morocco” OR “Mozambique” OR “Namibia” OR “Niger” OR “Nigeria” OR “Rwanda” OR “Sao Tome and Principe” OR “Senegal” OR “Seychelles” OR “Sierra Leone” OR “Somalia” OR “South Africa” OR “South Sudan” OR “Sudan” OR “Togo” OR “Tunisia” OR “Uganda” OR “United Republic of Tanzania” OR “Zambia” OR “Zimbabwe”))		643	06/11/2024 14:23:12
CENTRAL	#1	(“Africa” OR “Algeria” OR “Angola” OR “Benin” OR “Botswana” OR “Burkina Faso” OR “Burundi” OR “Cabo Verde” OR “Cameroon” OR “Central African Republic” OR “Chad” OR “Comoros” OR “Congo” OR “Côte d’Ivoire” OR “Democratic Republic of the Congo” OR “Djibouti” OR “Egypt” OR “Equatorial Guinea” OR “Eritrea” OR “Eswatini” OR “Ethiopia” OR “Gabon” OR “Gambia” OR “Ghana” OR “Guinea” OR “Guinea-Bissau” OR “Kenya” OR “Lesotho” OR “Liberia” OR “Libya” OR “Madagascar” OR “Malawi” OR “Mali” OR “Mauritania” OR “Mauritius” OR “Morocco” OR “Mozambique” OR “Namibia” OR “Niger” OR “Nigeria” OR “Rwanda” OR “Sao Tome and Principe” OR “Senegal” OR “Seychelles” OR “Sierra Leone” OR “Somalia” OR “South Africa” OR “South Sudan” OR “Sudan” OR “Togo” OR “Tunisia” OR “Uganda” OR “United Republic of Tanzania” OR “Zambia” OR “Zimbabwe”):ti, ab, kw	5	06/11/2024 16:09:53
	#2	(“Oral cancer” OR “Mouth cancer” OR “Mouth neoplasm” OR “Mouth tumor” OR “Oral neoplasm” OR “Oral tumor” OR “Oropharyngeal cancer” OR “Oropharyngeal neoplasm” OR “Tongue cancer” OR “Palatal cancer” OR “Cheek cancer” OR “Buccal cancer” OR “Floor of mouth cancer”):ti, ab, kw		
	#3	#1 AND #2		

## Abbreviations

CENTRAL: Cochrane Central Register of Controlled Trials
HIV: Human Immunodeficiency Virus
HNC: Head and Neck Cancer
ICD: International Classification of Diseases
JBI: Joanna Briggs Institute
MeSH: Medical Subject Headings
PRISMA-ScR: Preferred Reporting Items for Systematic Reviews and Meta-Analyses Extension for Scoping Reviews
ScR: Scoping Review
SEM: Socio-Ecological Model
UHC: Universal Health Coverage
